# Identification of the *Gossypium hirsutum* SDG Gene Family and Functional Study of *GhSDG59* in Response to Drought Stress

**DOI:** 10.3390/plants13091257

**Published:** 2024-04-30

**Authors:** Ziyu Wang, Wanwan Fu, Xin Zhang, Yunhao Liusui, Gulisitan Saimi, Huixin Zhao, Jingbo Zhang, Yanjun Guo

**Affiliations:** Xinjiang Key Laboratory of Special Species Conservation and Regulatory Biology, College of Life Science, XinjiangNormal University, Urumqi 830017, China; 18699315627@163.com (Z.W.); 15929146569@163.com (W.F.); zhang198616079@163.com (X.Z.); liusuiyunhao@163.com (Y.L.); 13345443145@163.com (G.S.); zhaohuixin101@sina.com (H.Z.)

**Keywords:** SDG, cotton, drought stress, functional study, transcriptomic

## Abstract

SET-domain group histone methyltransferases (SDGs) are known to play crucial roles in plant responses to abiotic stress. However, their specific function in cotton’s response to drought stress has not been well understood. This study conducted a comprehensive analysis of the SDG gene family in *Gossypium hirsutum*, identifying a total of 82 SDG genes. An evolutionary analysis revealed that the SDG gene family can be divided into eight subgroups. The expression analysis shows that some *GhSDG* genes are preferentially expressed in specific tissues, indicating their involvement in cotton growth and development. The transcription level of some *GhSDG* genes is induced by PEG, with *GhSDG59* showing significant upregulation upon polyethylene glycol (PEG) treatment. Quantitative polymerase chain reaction (qPCR) analysis showed that the accumulation of transcripts of the *GhSDG59* gene was significantly upregulated under drought stress. Further functional studies using virus-induced gene silencing (VIGS) revealed that silencing *GhSDG59* reduced cotton tolerance to drought stress. Under drought conditions, the proline content, superoxide dismutase (SOD) and peroxidase (POD) enzyme activities in the *GhSDG59*-silenced plants were significantly lower than in the control plants, while the malondialdehyde (MDA) content was significantly higher. Transcriptome sequencing showed that silencing the *GhSDG59* gene led to significant changes in the expression levels of 1156 genes. The KEGG enrichment analysis revealed that these differentially expressed genes (DEGs) were mainly enriched in the carbon metabolism and the starch and sucrose metabolism pathways. The functional annotation analysis identified known drought-responsive genes, such as ERF, CIPK, and WRKY, among these DEGs. This indicates that GhSDG59 is involved in the drought-stress response in cotton by affecting the expression of genes related to the carbon metabolism and the starch and sucrose metabolism pathways, as well as known drought-responsive genes. This analysis provides valuable information for the functional genomic study of SDGs and highlights potential beneficial genes for genetic improvement and breeding in cotton.

## 1. Introduction

In eukaryotic organisms, chromatin is a highly ordered complex composed of DNA and proteins. The basic repeating unit of chromatin is the nucleosome, which consists of DNA and histone proteins intertwined and distributed in specific spaces within the cell nucleus [1]. The nucleosome is an octamer composed of four core histones—H2A, H2B, H3, and H4—each of which is composed of two copies, and 147 bp core DNA wrapped around it [1]. Core histones can be modified by multiple covalent groups, mainly on their N-termini, including methylation, acetylation, phosphorylation, SUMOylation, ADP-ribosylation, ubiquitination, and a series of modifications [2,3]. These important histone modifications regulate gene expression by affecting the structure of chromatin, and they play an important regulatory role in many biological processes such as DNA replication, DNA repair, RNA transcription, growth and development, and stress response in plants [4]. Histone methylation is one of the most common types of histone modifications. This modification is dynamically regulated by “writer” enzymes called histone methyltransferases (HMTs) and “eraser” enzymes called histone demethylases (HDMs). The former is responsible for adding marks, while the latter removes marks. Subsequently, “reader” effector proteins that bind to histone modifications translate this information into transcriptional regulatory signals [4,5,6]. Current research has found that histone methylation primarily occurs on conserved arginine residues at the N-terminal tails of histones H3 and H4, including positions K4, K9, K27, K36, and K79 of histones H3 and H4 [7]. Histone arginine methylation is catalyzed by SET-domain group proteins (SDGs). Methylation of histone H3 can occur as mono-, di-, or trimethylation. Methylation at the H3K4 and H3K36 sites is associated with transcriptional activation, while methylation at the H3K9 and H3K27 sites is associated with transcriptional repression. In recent years, SDGs have been identified in various plants, including *Arabidopsis* [8], maize [9], rice [10], wheat [11], *Gossypium raimondii* [12], grapevine [13], tomato [14], poplar [15], citrus [16], *Dendrobium officinale* (traditional Chinese medicinal herb) [17], foxtail millet [18], and apple [19].

The SDG protein plays an important role in the growth and development of plants. In *Arabidopsis*, the SDG gene *AtATX1* specifically regulates the H3K4 trimethylation level of the FLOWERING LOCUS C (FLC) gene to promote its expression, thereby controlling the flowering time of Arabidopsis [20]. *AtSDG26* has been identified as a histone methyltransferase involved in flowering activation. Further analysis reveals that the loss of *SDG26* function leads to decreased levels of H3K4me3 and H3K36me3 modifications on its target gene *SOC1*, resulting in the suppression of gene expression. As a result, mutant plants with the *SDG26* mutation exhibit a phenotype of delayed flowering [21]. The rice *SDG712* gene acts as a negative regulator of flowering time. It negatively regulates the expression of key flowering genes *Ehd1* and flowering hormone genes *Hd3a* and *RFT1* by modulating their H3K9me2 levels, thus delaying the flowering time of rice [22]. *SDG708* is a positive regulatory factor for the flowering time of rice, which can regulate the expression of multiple flowering genes by controlling the levels of H3K36me1/2/3 modifications [23]. The overexpression of the *SUVH2 (AtSDG3*) gene in *Arabidopsis* delays leaf senescence, significantly upregulating the levels of H3K27me2 and H3K27me3 marks on the leaf senescence-related gene *WRKY53* in the overexpressing plants [24]. The *SUVH5/6* (*AtSDG9* and *AtSDG23*) in Arabidopsis plays a role in leaf development by regulating the H3k9me2 levels of the leaf development-related genes *KNAT1* and *KNAT2* [25]. ASHR3 (SDG4) regulates cell division in the root apical meristem of Arabidopsis. The lengths of the root apical meristem and primary root are shorter in the ashr3 mutant compared to the wild type [26]. SDG2 plays an important role in maintaining the primary root stem cell niche, as well as establishing the lateral root stem cell niche. Loss of SDG2 function results in a significant reduction in H3K4me3 levels in the root stem cell niche and differentiated cells, and leads to the loss of the auxin gradient maximum in the root quiescent center [27]. AtATX1 (SDG27) plays a crucial role in the establishment of root system architecture, as it is involved in the timing control of root development, maintenance of the stem cell niche, and cell patterning during primary and lateral root development [28]. *AtATX4 (SDG16)* is involved in shoot identity establishment by conferring H3K4me3 deposition at the loci of shoot identity genes [29]. SDG711 is a histone H3K27me2/3 methyltransferase in rice, which is homologous to CLF in *Arabidopsis*. Mutants of SDG711 result in increased gene expression levels related to starch accumulation and decreased levels of H3K27me3, leading to smaller seeds and even sterility. SDG711 also plays a crucial role in regulating flowering time and panicle development [30]. NtSET1 in tobacco has been shown to bind to chromatin and possess H3K9 histone methyltransferase activity. Its ectopic expression enhances H3K9 methylation, affecting chromatin segregation in tobacco suspension culture cells and suppressing plant growth [31].

The SDG protein also plays an important role in plant responses to abiotic stress. *ATX1* is a positive regulatory factor in Arabidopsis’s response to drought stress. Drought stress promotes the binding of *ATX1* to *NCED3*, and *ATX1* enhances the expression of *NCED3* by increasing the H3K4me3 level [32]. *CsSDG36* is an H3K4 methyltransferase, and overexpression of *CsSDG36* reduces the tolerance of *Arabidopsis* to hyperosmotic stress [33]. Transgenic plants overexpressing *OsSDG721* showed saline–alkaline stress-tolerant phenotypes. *OsSDG721* can deposit the H3K4me3 mark in the promoter and coding regions of *OsHKT1;5*, thereby increasing the expression of *OsHKT1;5* under saline–alkaline stress [34]. *OsSDG708* functions as a positive regulator in the drought response of rice, Os*SDG708* can promote ABA biosynthesis by activating the expressions of *OsNCED3* and *OsNCED5* [35]. *OsERF103* recruits *OsSDG705* to enhance the H3K4me3 modification level of the drought-responsive gene *OsbZIP23*, thereby promoting the expression level of *OsbZIP23* under drought stress [36]. In tomato, *SDG33* and *SDG34* are associated with drought response, and the *sdg33/34* double mutant exhibits stronger tolerance to drought stress [37]. H3K4 methyltransferases SDG25 and ATX1 are required for heat-stress tolerance in *Arabidopsis*, and they can maintain the expression of the stress response during heat stress [38]. In foxtail millet, *SiSET14* is upregulated by cold stress, and the overexpression of *SiSET14* enhances yeast’s tolerance to cold stress [18].

Cotton is a globally important economic crop and plays a crucial role in socioeconomic development. Upland cotton (*Gossypium hirsutum*) is the largest cultivated tetraploid cotton variety in the world and also the largest natural fiber source crop, accounting for over 95% of global cotton production [39]. With the increase in global climate change and extreme weather events, drought has become the most common abiotic stress in major cotton-producing regions, severely impacting the growth, development, yield, and quality of cotton. Therefore, exploring the molecular mechanisms of cotton’s response to drought and discovering drought-resistant gene resources in cotton will contribute to the breeding of more drought-tolerant cotton varieties. Previous studies have shown that the SDG gene family plays an important role in the plant’s response to environmental stress [12,18,19]. Jian et al. also reported the functional role of an SDG gene, *GhSDG51*, in the salt stress response of cotton. However, the specific function of SDG genes in cotton’s response to drought is still unclear [40].

In this study, we identified the cotton SDG gene family members at the whole-genome level and analyzed their evolutionary relationship. The phylogenetic tree indicates that cotton SDG genes are subdivided into eight subgroups. The expression patterns of SDG genes were investigated in different tissues of cotton and under stress conditions. Transcriptome and qPCR analyses revealed that the expression of *GhSDG59* was significantly induced by PEG treatment and drought stress. Furthermore, we used VIGS technology to investigate the function of the *GhSDG59* gene. We found that silencing *GhSDG59* resulted in reduced tolerance of cotton to drought stress. Transcriptome sequencing analysis was conducted on the control and *GhSDG59* gene-silenced cotton plants, revealing that the expression of 1156 genes was altered in the *GhSDG59* gene-silenced plants. These differentially expressed genes were mainly enriched in the carbon metabolism and the starch and sucrose metabolism pathways, and they included several known drought-responsive genes. This study expands our understanding of the function of plant SDG genes and provides a basis for further investigating the role and regulatory mechanisms of SDG genes in cotton’s response to drought stress.

## 2. Results

### 2.1. Identification and Phylogenetic Analysis of the Gossypium hirsutum SDG Gene Family

**Figure 1:**
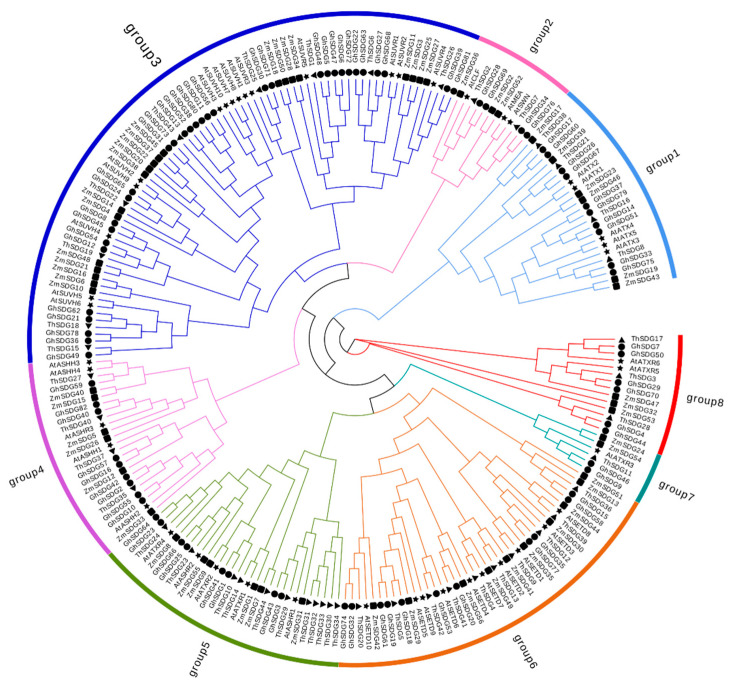
Phylogenetic relationships of SDG proteins from *Gossypium hirsutum*, *Theobroma, Arabidopsis*, and maize. The unrooted phylogenetic tree was constructed using FastTree by maximum likelihood method, and the bootstrap test was performed with 1,000 iterations. The eight subgroups are indicated with different colors. Different shapes represent genes from different plant species, with the triangle, square, pentagon, and circle representing SDG genes from *Theobroma*, maize, *Arabidopsis*, and *Gossypium hirsutum*, respectively.

Following rigorous evaluation using BLASTP and HMMER 3.0 software programs, we ultimately identified 82 members of the SDG gene family from the *Gossypium hirsutum* whole genome. On the basis of their positions on the chromosomes, we named them *GhSDG1* to *GhSDG82*. The analysis of the SDG family protein characteristics showed significant variation in their amino acid lengths, with most falling between 362 and 969 amino acids. A small portion of proteins had lengths exceeding 1000 amino acids. Among them, GhSDG72 had the fewest amino acid residues, consisting of 150 aa, while GhSDG9 was the largest, containing 2427 aa. The molecular weights of the SDG proteins ranged from 16,730.86 to 276,053.45 Da, with theoretical isoelectric points ranging from 4.47 to 9.62. Subcellular localization prediction was conducted for the 82 *GhSDG* genes, revealing that the majority of the gene family members were found in the cytoplasm, nucleus, chloroplasts, and mitochondria (Table S1). Specifically, 68.29% of *GhSDG* genes (56 genes) were predicted to localize in the nucleus, while 24.39% (20 genes) were found in the chloroplasts. The remaining genes were located in the cytoplasm or mitochondria. Except for GhSDG23 and GhSDG59, the majority of SDG proteins are hydrophilic. To elucidate the phylogenetic relationships of *GhSDG*, a total of 228 SDG protein sequences from *Gossypium hirsutum*, Theobroma, Arabidopsis, and maize (*Zea mays*) were used to construct a phylogenetic tree using the maximum likelihood method (Figure 1). Evolutionary analysis has shown that SDG proteins can be categorized into eight distinct subgroups, namely, Groups 1 to 8. The number of SDG proteins varies significantly across these subgroups. Group 3 is the largest subgroup, comprising 76 SDG proteins. In contrast, Group 7 contains only six SDG protein members. Group 1, Group 3, Group 4, Group 5, Group 6, and Group 8 contain 28, 12, 24, 29, 41, and 15 SDG proteins, respectively. *GhSDG* genes are distributed in all subgroups. Among them, Group 3 has the highest number of GhSDG members, with 32 SDG proteins. This suggests that gene expansion events may have occurred in Group 3 during the evolution of *Gossypium hirsutum*. Conversely, Group 7 has the lowest number of *GhSDG* members, containing only two members. Numerous SDG genes of Arabidopsis have multiple homologous genes in *Gossypium hirsutum*. For instance, *GhSDG7* and *GhSDG50* serve as homologous genes for *AtATXR6*, while *GhSDG29* and *GhSDG70* are the homologous genes for *AtATXR5*. This is because the Gossypium hirsutum is an allopolyploid formed by the hybridization of two diploid cotton species, with most of the genes in Gossypium hirsutum being a pair of homologous genes corresponding to AA and DD, thus most *Arabidopsis* genes have two corresponding homologous genes in *Gossypium hirsutum* (Figure 1).

### 2.2. Analysis of the Expression Patterns of the SDG Gene Family Members in Different Tissues

**Figure 2:**
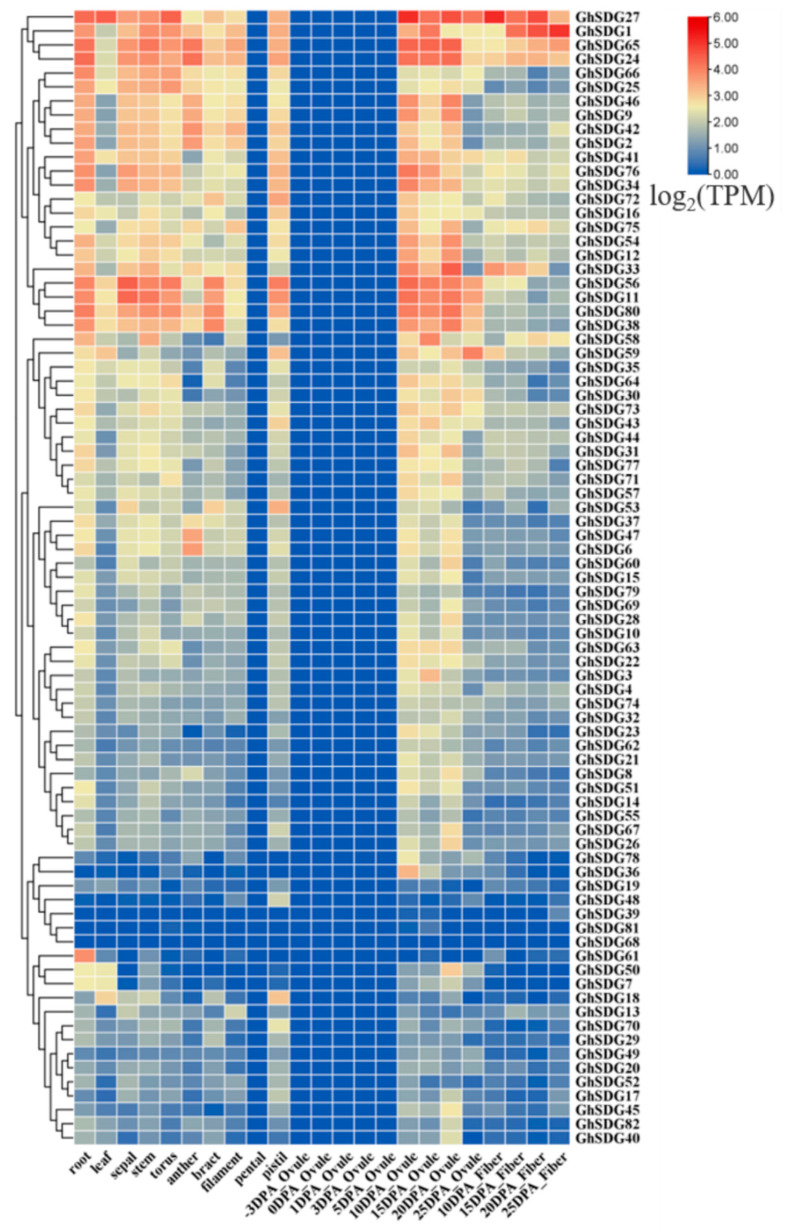
Expression analysis of *GhSDG* genes in different tissues and at different developmental stages of ovule and fiber in *Gossypium hirsutum*.

To explore the potential biological functions of the *GhSDG* genes in the growth and development of cotton, we downloaded the expression data of 12 organs, including roots, stems, leaves, sepals, receptacles, anthers, bracts, stigmas, petals, pistils, ovules, and fibers, from the CottonMD transcriptome database of cotton TM-1 “https://yanglab.hzau.edu.cn/CottonMD” (accessed on 13 October 2023) to analyze the expression patterns of the *GhSDG* gene family. The developmental stages of cotton ovules and fibers are described in days post anthesis (DPA). The analysis results indicate that *GhSDG*s are expressed in multiple tissues and exhibit different expression levels in different tissues and developmental stages, suggesting the involvement of *GhSDG* genes in cotton development (Figure 2). *GhSDG27*, *GhSDG24*, *GhSDG65*, and *GhSDG80* are predominantly expressed in roots, indicating their involvement in the development of cotton roots. The expression levels of *GhSDG27*, *GhSDG59*, *GhSDG18*, and *GhSDG56* are higher in the leaves, indicating their correlation with leaf development. Transcripts of *GhSDG11* and *GhSDG56* accumulate significantly in sepal and stem, implying their association with sepal and stem development. Additionally, some SDG genes are highly expressed in other tissues, such as *GhSDG27* and *GhSDG56* in torus, *GhSDG24* and *GhSDG65* in anther, *GhSDG38* and *GhSDG56* in bract and pistil. During ovule development, all *GhSDG* genes are not expressed in ovules from -3DPA to 5DPA, and in the late stage of ovule development, the expression of some *GhSDG* genes begins to upregulate. For instance, *GhSDG27*, *GhSDG65*, *GhSDG24*, *GhSDG56*, *GhSDG11*, and *GhSDG80* all show higher expression levels in ovules from 15DPA to 25DPA, indicating their involvement in the late development of ovules. *GhSDG31*, *GhSDG58*, and *GhSDG79* exhibit high expression in fibers at 10, 20, and 25 DPA, suggesting their roles in secondary cell wall biosynthesis of cotton fibers. Furthermore, genes like *GhSDG17*, *GhSDG31*, *GhSDG58*, and *GhSDG79* have high expression levels in multiple tissues, indicating their involvement in the development processes of multiple tissues. It is also noteworthy that all *GhSDG*s genes are almost not expressed in petals, indicating that *GhSDG* may not participate in petal development (Figure 2).

### 2.3. Expression Analysis of the GhSDG Gene Family under PEG and Drought Treatment Conditions

**Figure 3:**
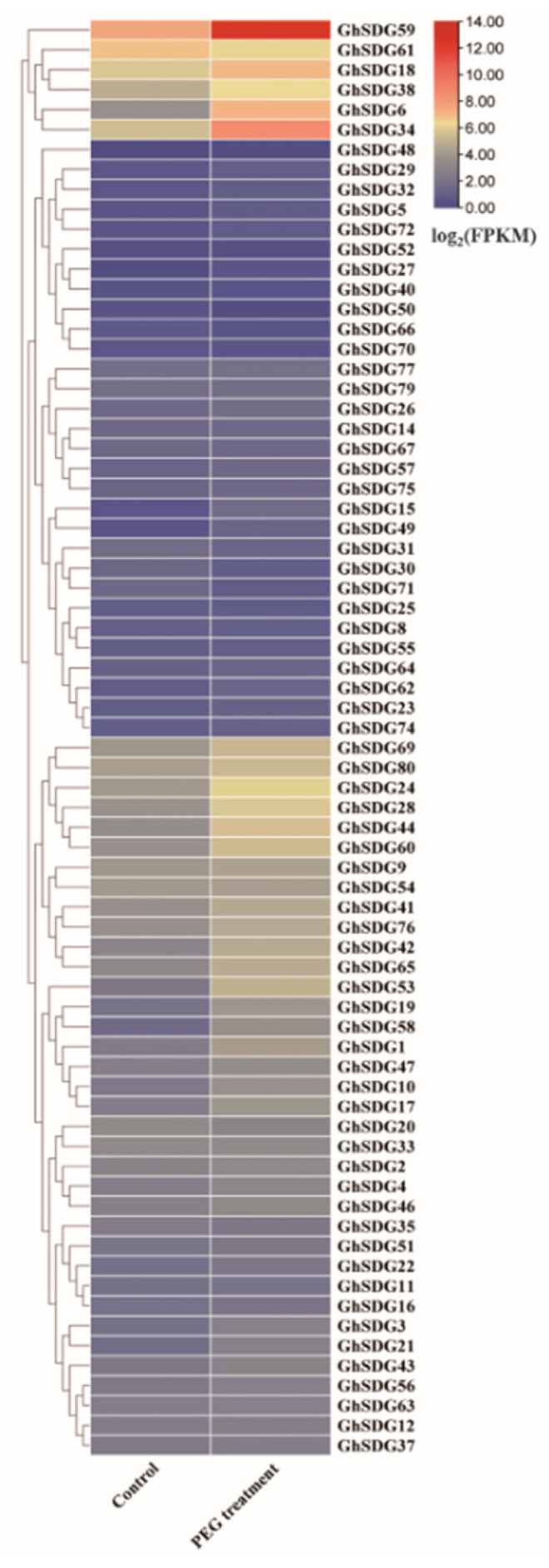
Expression pattern of *GhSDG* genes under PEG treatment conditions.

To investigate whether members of the *GhSDG* gene family are involved in the response of cotton to drought stress, we analyzed the expression patterns of *GhSDG* using transcriptome data from *Gossypium hirsutum* leaves under PEG treatment conditions. The expression analysis results show that some *GhSDG* genes did not exhibit significant differences between the control group and the PEG treatment group. However, the expressions of 25 *GhSDG* genes were upregulated under PEG treatment conditions (fold change > 1.5), among which we found that the expression of the *GhSDG59* gene was significantly induced by PEG treatment, indicating that the *GhSDG59* gene can respond to PEG treatment (Figure 3). The cis-acting element analysis showed the presence of ABRE and MBS cis-acting elements associated with drought stress in *GhSDG59* gene promoter, suggesting that it may be involved in plant response to drought (Figure S1 and Table S2).

To further ascertain the involvement of the *GhSDG59* gene in the response of cotton to drought stress, we assessed the expression of the *GhSDG59* gene under conditions of normal watering and drought treatment. Our qPCR analysis demonstrated a significant upregulation of the *GhSDG59* gene expression under drought stress conditions compared to normal watering conditions (Figure S2). This finding provides additional evidence supporting the involvement of the *GhSDG59* gene in the cotton plant’s response to drought stress.

### 2.4. Silencing GhSDG59 by VIGS Reduced the Drought Tolerance of Cotton

**Figure 4:**
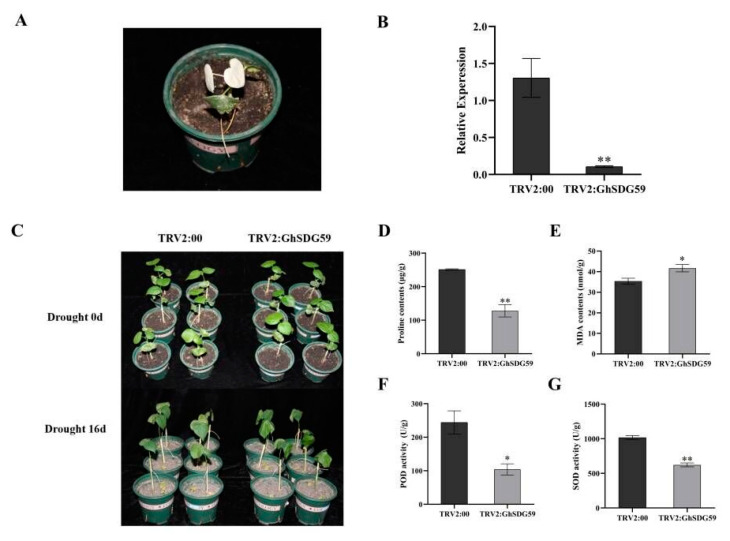
Silencing of *GhSDG59* compromised cotton tolerance to drought stress: (**A**) positive control plants; (**B**) expression of *GhSDG59* in TRV2:00 and TRV2*:GhSDG59* plants, and SDG represents the standard deviation derived from three separate trials; (**C**) phenotype of TRV2:00 and TRV2:*GhSDG59* plants under drought stress, and photographs were captured following 16 days of drought treatment; (**D**–**G**) proline content, MDA content, POD activity, and SOD activity of TRV2:00 and TRV2:*GhSDG59* plants under drought stress. The asterisks indicate significant differences according to the Student’s t-test. * *p* < 0.05; ** *p* < 0.01.

To explore the role of *GhSDG59* in cotton’s response to drought stress, we utilized virus-induced gene silencing (VIGS) technology to suppress the expression of *GhSDG59*. As depicted in Figure 4A, one week post injection of the Agrobacterium suspension, the leaves of the TRV2:*GhCLA1* cotton plants displayed a whitening phenotype, indicating successful suppression of the target gene’s expression (Figure 4A). To further validate the effective silencing of *GhSDG59*, we employed qPCR to measure the expression levels of *GhSDG59* in TRV2:00 and *TRV2*:*GhSDG59* cotton plants. The results demonstrate a significant reduction in the expression level of the *GhSDG59* gene in the TRV2:*GhSDG59* plants compared to the TRV2:00 plants (*p* < 0.01), confirming the successful inhibition of the *GhSDG59* gene expression in cotton plants (Figure 4B). Subsequently, we conducted a comparative analysis of the phenotypes between the control and gene-silenced cotton plants. Under optimal growth conditions, there were no discernible differences in phenotype between the TRV2:00 and TRV2:*GhSDG59* cotton plants. However, following a 16-day period of natural drought, the wilting severity of the leaves in the TRV2:*GhSDG59* cotton plants was significantly higher compared to the TRV2:00 plants (Figure 4C).

Furthermore, we examined the physiological parameters of cotton leaves under drought treatment. The results show that under drought stress conditions, the PRO content in the TRV2:*GhSDG59* cotton plants was significantly lower than that in the TRV2:00 plants, indicating that silencing the *GhSDG59* gene reduced the osmotic potential of cotton under drought stress conditions (Figure 4D). Drought stress led to a higher accumulation of MDA in the TRV2:*GhSDG59* cotton plants, suggesting a higher level of damage compared to the TRV2:00 cotton plants (Figure 4E). After drought treatment, the SOD and POD enzyme activities in the TRV2:00 cotton plants were higher than those in the TRV2:*GhSDG59* cotton plants, indicating that inhibiting the expression of the *GhSDG59* gene reduced the antioxidant capacity of cotton plants (Figure 4F,G). The phenotypic analysis and physiological parameter measurements all indicate that silencing the *GhSDG59* gene reduces the tolerance of cotton plants to drought stress.

### 2.5. Transcriptome Analysis of TRV2:00 and TRV2:GhSDG59 Cotton Plants under Drought Stress

**Figure 5:**
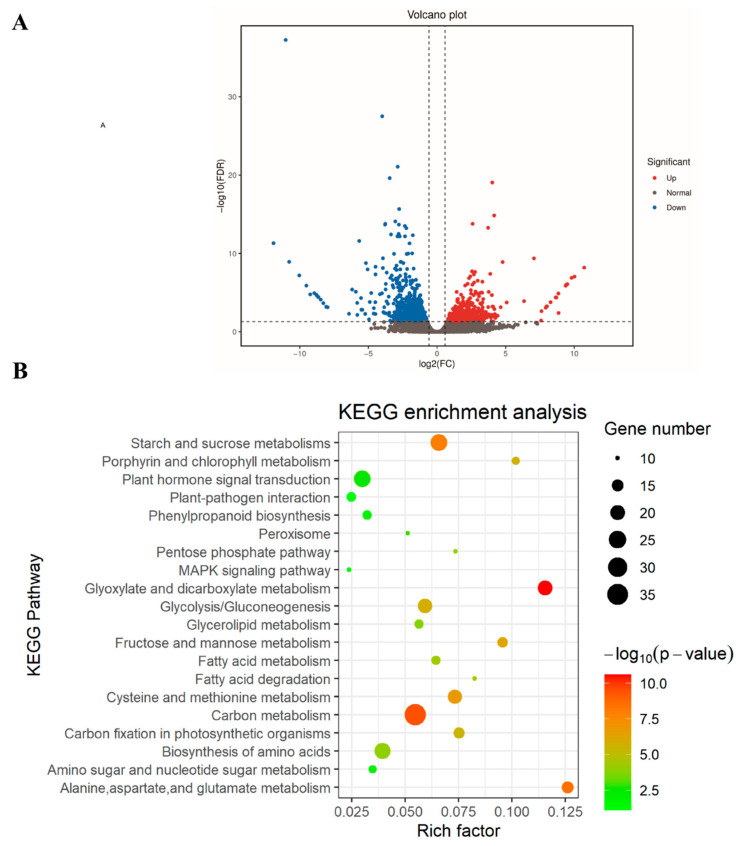
Transcriptome sequencing analysis of TRV2:00 and TRV2:*GhSDG59* plants under drought stress: (**A**) volcano plot showing the distribution of DEGs. Red and blue dots represent up and downregulated genes, respectively; (**B**) KEGG enrichment analysis of DEGs. The enrichment factor represents the ratio of the proportion of differentially expressed proteins to the proportion of total *Gossypium hirsutum* proteins annotated as involved in a given pathway.

To further investigate the genes and pathways influenced by *GhSDG59* under drought stress conditions, we performed transcriptome sequencing on the leaves of TRV2:00 and TRV2:*GhSDG59* cotton plants. Under drought stress conditions, the expression of 482 genes was upregulated in TRV2:*GhSDG59* cotton plants compared to TRV2:00 cotton plants, while the expression of 674 genes was downregulated in TRV2:*GhSDG59* cotton plants compared to TRV2:00 cotton plants (Figure 5A).

**Figure 6:**
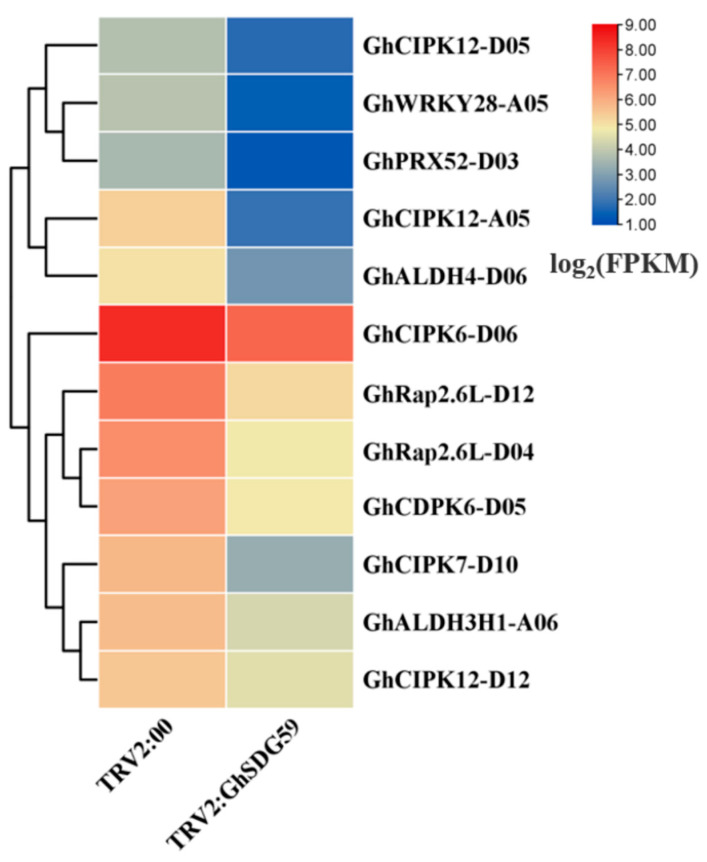
Expression analysis of drought-responsive genes in TRV2:00 and TRV2:*GhSDG59* plants under drought stress.

A further enrichment analysis was conducted on the differentially expressed genes obtained using Gene Ontology (GO) and the Kyoto Encyclopedia of Genes and Genomes (KEGG). The Gene Ontology enrichment assessment indicates that DEGs are predominantly overrepresented in cellular constituents, such as chloroplast, plastid, chloroplast stroma, and the cell wall (Figure S3A). The molecular functions primarily annotated for DEGs through GO enrichment analysis are hydrolase activity, hydrolyzing O-glycosyl compounds, protochlorophyllide reductase activity, and pyridoxal phosphate binding (Figure S3B). The biological processes that DEGs are primarily involved in include carbohydrate metabolic process, photosynthesis, and auxin-activated signaling pathway (Figure S3C). The KEGG enrichment analysis indicates that the DEGs primarily participate in metabolic and signaling pathways, including the carbon metabolism, starch and sucrose metabolisms, glyoxylate and dicarboxylate metabolism, and glycolysis/gluconeogenesis (Figure 5B). Further, we found that multiple known drought response genes are present in the DEGs, including WRKY, ERF, CIPK, and ALDH (acetaldehyde dehydrogenase) (Figure 6).

## 3. Discussion

With the release of more and more plant genome data, many gene families have been identified and analyzed in different plants, and previous studies have shown that some gene families play crucial roles in responding to drought stress in plants [41,42,43,44]. The SDG gene family is a class of histone lysine methyltransferases that play important roles in plant growth, development, and response to environmental stress. Zhang et al. analyzed the SDG genes from 145 sequenced species, including 34 plants and 59 animals, and found that, on average, plants contain 47 SDG genes. In this study, we identified 82 SDG gene family members in the *Gossypium hirsutum* genome, which is higher than in other plants, such as *Arabidopsis* (33) [8] and *apple* (67) [19], suggesting a greater potential for further exploration of SDG genes in *Gossypium hirsutum*.

SDG genes play important roles in plant growth and development. For example, the SDG gene *ASHR3C* in *Arabidopsis* is involved in stamen development [45]. The *AtSDG26* gene regulates trichome development in *Arabidopsis*, and mutation of this gene increases the number of trichomes on rosette leaves [46]. *OsSDG711* affects the size of rice organs by regulating cell length and width [47]. The MdSDG gene family members are highly expressed in different tissues and at different stages of fruit development in apple, indicating their involvement in apple growth and development [48]. An analysis of tissue-specific expression of the *GhSDG* gene family in *Gossypium hirsutum* showed that members of the *GhSDG* gene family are dominantly expressed in multiple tissues and at different developmental stages of ovules and fibers, indicating their involvement in upland cotton’s growth and development.

Previous research has shown that members of the SDG gene family are involved in the response of plants to abiotic stress [48]. The SDG genes in apple can respond to cold, heat, and drought stress [19]. The expression of multiple *ZmSDG* genes can be altered by PEG or salt stress. The expression of the *Dendrobium catenatum* SDG gene can be induced by drought, low temperature, and high temperature [17]. Overexpression of the tea *CsSDG36* gene reduces the tolerance of *Arabidopsis* to osmotic stress [33]. Histone 3 lysine methyltransferases SDG33 and SDG34 are negative factors in the tomato’s drought response, with the *SDG33/SDG34* double mutant exhibiting increased tolerance to drought stress [49]. The promoter of *GhSDG51* contains drought stress-related *cis*-acting elements, and its expression is significantly induced by salt stress; further silencing of this gene has been found to reduce the tolerance of cotton to salt stress [40]. Our research found that the expression of a total of 44 *GhSDG* genes changed under PEG treatment conditions, indicating that the GhSDG gene family is involved in the drought response of cotton. Among these 44 *GhSDG* genes with altered expression, we found that the expression of *GhSDG59* was significantly induced by PEG treatment, and the qPCR analysis showed that the expression of *GhSDG59* was also induced by drought stress. Additionally, its promoter contains multiple drought response related *cis*-acting elements; therefore, we conducted further studies on the function of this gene. The function verification indicates that silencing the *GhSDG59* gene reduces the tolerance of cotton to drought stress. Our results further confirm the important role played by members of the SDG gene family in plant responses to abiotic stress.

Proline is an important osmotic regulatory substance. Drought stress increases the proline content in plants, which helps maintain the plants’ osmotic regulatory ability and reduces lipid peroxidation to preserve cell membrane integrity [50]. In our research, we found that under drought stress conditions, the proline content of TRV2:*GhSDG59* plants was lower than that of TRV2:00 plants, while the MDA content was higher than that of TRV2:00 plants. This indicates that silencing *GhSDG59* reduces the plant’s osmotic regulation ability, leading to lipid peroxidation and damage to the cell membrane. Drought stress can lead to the accumulation of a large amount of ROS (reactive oxygen species) in plant cells, oxidizing biomolecules and hindering normal metabolism and growth, even causing plant death [50]. In order to reduce the toxicity of ROS, plants have evolved an efficient antioxidant system to eliminate excess ROS and maintain healthy growth. SOD and POD are two important antioxidant enzymes, and plants under drought stress can reduce ROS levels by increasing the activities of these enzymes [51]. Under drought stress conditions, the activities of SOD and POD enzymes in *GhSDG59* gene silenced plants were lower than those in control plants. This indicates that inhibiting the expression of the *GhSDG59* gene reduces cotton’s ability to remove ROS, leading to more severe cell damage.

SDG proteins play a role in plant responses to abiotic stress by controlling the expression of downstream genes [48]. In rice, *OsSDG708* acts as a positive regulator of drought response. The expression of 702 genes decreased in *OsSDG708* RNAi plants and increased in *OsSDG708* overexpressing plants. Among these 702 genes, there are several known drought response genes, indicating that *OsSDG708* can regulate rice’s response to drought by modulating the expression of drought-responsive genes [35]. *CsSDG36* overexpression reduces the tolerance of transgenic *Arabidopsis* to osmotic stress and also affects the expression of genes related to the chromatin assembly, microtubule assembly, and leaf stomatal development pathways [33]. Our research shows that silencing *GhSDG59* leads to changes in the expression of 1156 genes, including multiple known drought-response genes, suggesting that *GhSDG59* also participates in the cotton response to drought stress by influencing the expression of drought-response genes.

In *Masson Pine*, drought stress induced changes in the expression of multiple genes related to starch and sucrose metabolisms, indicating the involvement of these genes in the *Masson Pine*’s response to drought stress [52]. *ZmSUS1* enhances maize tolerance to drought stress by regulating the expression of genes involved in sucrose metabolism [53]. The transcription factor *ScDREB10* can regulate the expression of genes in the starch and sucrose metabolism pathways to enhance plant stress tolerance [54]. Potassium ions alleviate drought damage by regulating sucrose metabolism in sesame leaves [55]. In switchgrass, drought stress induces significant changes in the expression of genes related to starch and sucrose metabolism pathways [56]. When Sugarcane Saccharumzai is subjected to drought stress, the transcription levels of numerous genes related to starch and sucrose metabolisms are altered [57]. The enrichment analyses of GO and KEGG in this study demonstrate that the 74 DEGs are mainly related to carbon metabolism and the pathways of starch and sucrose metabolisms, suggesting that *GhSDG59* can also enhance cotton’s tolerance to drought stress by modulating the expression of genes involved in the carbon metabolism and the starch and sucrose metabolism pathways. Both our results and those of previous studies have confirmed the significant role of starch and sucrose metabolism pathways in plants responding to drought stress. The above results have expanded our understanding of the regulatory mechanism of plant SDG genes.

## 4. Materials and Methods

### 4.1. Identification and Physicochemical Property Analysis of SDG Gene Family Members in Cotton

To identify the members of the SDG gene family in cotton, the hidden Markov model (PF00221) of SDG protein domains was first downloaded from the pfam3.0 database “https://www.ebi.ac.uk/interpro/wwwapi//entry/pfam/PF00221?annotation=hmm” (accessed on 10 October 2023). The genome sequence and protein sequence of upland cotton (TM1_WHU) were then downloaded from the CottonMD database “https://yanglab.hzau.edu.cn/CottonMD” (accessed on 13 October 2023). HMMER 3.0 software was used with the hidden Markov model of SDG protein domains as a probe to search for and identify the members of the SDG gene family in the cotton genome. TBtools (v1.123) software was used to analyze the basic physicochemical properties of cotton SDG proteins, such as molecular weight, isoelectric point, and amino acid quantity. The subcellular localization of cotton SDG proteins was predicted using WoLF PSORT “http://wolfpsort.seq.cbrc.jp” (accessed on 14 October 2023).

### 4.2. Phylogenetic Analysis of SDG Genes

First, we performed multiple sequence alignment on the SDG protein sequences originating from *Gossypium hirsutum* and other plant species. Then, we input the results of the multiple sequence alignment into the MEGA11 software to construct a phylogenetic tree using the maximum likelihood method, with the parameter set to bootstrap for 1000 times.

### 4.3. Expression Analysis of the GhSDG Gene Family

Transcriptome data of various tissues, ovules, and fibers during development in Gossypium hirsutum cotton were downloaded from the CottonMD database “https://yanglab.hzau.edu.cn/CottonMD” (accessed on 13 October 2023) and then the expression data were used to generate heat maps using TBtools (v1.123) software [58,59]. The expression data under the PEG treatment conditions came from our laboratory, and this set of data is from the transcriptome sequencing of cotton leaves after 18% PEG treatment.

### 4.4. Analysis of Cis-Acting Elements in the GhSDG59 Gene Promoter

The 2000bp sequence upstream of the *GhSDG59* gene start codon was extracted from the *Gossypium hirsutum* genome as the promoter sequence. The online software PlantCARE “http://bioinformatics.psb.ugent.be/webtools/PlantCare/html/” (accessed on 16 October 2023) was used to predict the cis-acting elements present in the promoter. The results of the promoter analysis were then visualized using Tbtools software (v1.123).

### 4.5. Plant Material Cultivation

The cotton seeds of XinluZao 42 were generously provided by the Cotton Germplasm Resources Research Office of the Xinjiang Academy of Agricultural Sciences in China. The cotton seeds were germinated on moist filter paper and then planted in pots containing a mixture of nutrient soil and vermiculite (at a ratio of 3:1 by volume). The soil in each flower pot was preweighed to ensure a consistent soil volume in each pot. The cotton seedlings were cultivated in a greenhouse at a temperature of 25–28 °C and a light cycle of 16 h light/8 h dark.

### 4.6. RNA Extraction and Real-Time Fluorescence Quantitative PCR (qRT-PCR) Analysis

According to the manufacturer’s instructions, the total RNA from cotton leaves was extracted using the Plant Total RNA Isolation Kit Plus (FOREGENE, Chengdu, China). The quality and concentration of the RNA samples were assessed using 1% agarose gel electrophoresis, and a NanoDrop 2000 spectrophotometer (Thermo Fisher Scientific, Wilmington, DE, USA). The extracted RNA was reverse transcribed into cDNA using the FOREGENE (Chengdu, China) Master Premix (For qPCR) RT EasyTM II reverse transcription kit. Real-time fluorescence quantitative PCR was performed using the Quant Gene 9600 system (Bioer Technology, Hangzhou, China) with the following reaction conditions: 95 °C for 3 min, followed by 40 cycles of 95 °C for 10 s, 58 °C for 10 s, 72 °C for 20 s, 95 °C for 15 s, 58 °C for 1 min, and 95 °C for 15 s. The *GhHis3* gene of cotton was used as an internal reference gene, and the expression level of the target gene was calculated using the 2^−ΔΔCt^ method. All analyses were performed in triplicate biological and technical replicates. The primers used in this study are listed in Supplementary Table S3.

### 4.7. VIGS Experiment

The 400bp specific *GhSDG59* gene fragment was inserted into TRV2 to construct the TRV2*-GhSDG59* vector. The TRV1, TRV2, and TRV2:*GhSDG59* vector was transferred into Agrobacterium, and the Agrobacterium liquid was injected into cotton cotyledons according to the methods described by previous researchers [60]. The cotton injected with the mixed Agrobacterium liquid of TRV1 and TRV2 was the control plant (TRV2:00), and the cotton injected with the mixed Agrobacterium liquid of TRV1 and TRV2*:GhSDG59* was the gene-silencing plant (TRV2:*GhSDG59*). The expression of *GhSDG59* in TRV2:00 and TRV2:*GhSDG59* cotton plant was detected through qPCR. The cotton plants with effective silencing of *GhSDG59* gene were used for further analysis.

### 4.8. Drought Treatment Experiment

Twelve pots of 3-week-old cotton plants with the same growth status of TRV2:00 and TRV2:*GhSDG59* were used for drought treatment. Before the drought treatment, 200 mL of water was poured into the pot which contained the same amount of soil, and then the watering was stopped for natural drought treatment. We took pictures after the TRV2:00 and TRV2:*GhSDG59* cotton plants showed phenotypic differences.

### 4.9. Determination of Physiological Indicators

The absolute soil water content (ASWC) was used to assess the level of drought the cotton plants experienced. The calculation formula for the ASWC (%) is (WW1 − (weight of dried mixed nutrient soil + weight of pot))/weight of dried mixed nutrient soil × 100. Moderate drought refers to an ASWC of around 10%. When the TRV2:00 cotton plants reach moderate drought, cotton leaves are collected for physiological indicators determination. The contents of proline and MDA were determined according to the instructions provided with the MDA detection kit and proline detection kit (Nanjing Jiancheng Bioengineering Institute, Nanjing, China). We determined the SOD and POD enzyme activities in the leaves according to the experimental methods provided in the instructions for the SOD and POD detection kit (Nanjing Jiancheng Bioengineering Institute, Nanjing, China).

### 4.10. RNA-Seq Analysis

When the TRV2:00 cotton plants reached a moderate drought, the cotton leaf RNA was extracted for transcriptome sequencing. To assess the purity and concentration of RNA, the NanoDrop 2000 spectrophotometer was used, followed by the evaluation of the RNA integrity using the Agilent 2100/LabChip GX. Once the quality of the RNA samples was confirmed, we proceeded with the library construction by following these steps: (1) enrich eukaryotic mRNA with Oligo(dT) magnetic beads; (2) fragment the mRNA with Fragmentation Buffer; (3) synthesize the first and second strands of cDNA using the mRNA as a template and purify the cDNA; (4) perform end repair, A-tailing, and adapter ligation on the purified double-stranded cDNA, followed by fragment size selection using AMPure XP beads; and (5) enrich the cDNA library through PCR. Finally, performed paired-end 150 (PE150) sequencing on the quality-checked cDNA library using an Illumina NovaSeq 6000 sequencing platform.

Upon completion of the sequencing process, the bioinformatics analysis was carried out using the provided analysis pipeline from BMKCloud “www.biocloud.net” (accessed on 21 February 2024). Firstly, we evaluated the quality of the sequencing data and then filtered out the low-quality sequencing data. We aligned the sequencing data to the *Gossypium hirsutum* reference genome. We performed gene expression quantification analysis on the obtained transcripts. Subsequently, differential gene expression screening was conducted using a fold change ≥ 1.5 and an FDR < 0.05 as the screening criteria. We utilized the BMKCloud and KOBAS tools “http://bioinfo.org/kobas” (accessed on 24 February 2024) for the GO and KEGG enrichment analyses of the DEGs [61].

## 5. Conclusions

In this study, we identified 82 SDG genes in the cotton genome. The evolutionary analysis revealed that the SDG gene family can be divided into eight subgroups. The expression analysis indicated that the SDG genes are predominantly expressed in multiple cotton tissues, suggesting their involvement in cotton growth and development processes. Transcriptome data under PEG treatment conditions showed altered expression of multiple SDG genes, with the *GhSDG59* gene significantly induced by PEG treatment. Subsequent qPCR analysis of the *GhSDG59* gene’s expression under drought stress demonstrated significant induction in response to drought stress, further supported by transcriptome data and qPCR analysis under PEG treatment conditions, highlighting the involvement of the *GhSDG59* gene in cotton’s response to drought stress. Silence of the *GhSDG59* gene using VIGS technology resulted in reduced drought tolerance in cotton plants. Under drought stress conditions, the proline content and SOD and POD enzyme activities in cotton plants with the silenced *GhSDG59* gene were lower than those in the control plants, while the MDA content was higher. Transcriptome analysis showed that silencing the *GhSDG59* gene altered the expression of 1156 genes, mainly enriched in the carbon metabolism and the starch and sucrose metabolism pathways. Among these differentially expressed genes were known drought-responsive genes, such as WRKY, ERF, CIPK, and ALDH, indicating that the *GhSDG59* gene regulates the expression of genes related to the carbon metabolism and the starch and sucrose metabolism pathways, as well as some known drought-responsive genes, contributing to cotton’s defense against drought stress. Our study lays the foundation for further investigation into the function and mechanism of the *GhSDG59* gene in cotton’s response to drought stress.

## Data Availability

Transcriptome data involved in this study can be obtained from the corresponding author (18910445207@163.com) upon reasonable request.

## Supplementary Materials

The following supporting information can be downloaded at: https://www.mdpi.com/article/10.3390/plants13091257/s1, Figure S1: Analysis of *cis*-acting elements on the *GhSDG59* gene promoter; Figure S2: Expression analysis of the *GhSDG59* gene under normal watering and drought stress; Figure S3: Gene Ontology annotation analysis of DEGs; Table S1: Characteristics of SDG family genes in *Gossypium hirsutum*; Table S2: Function annotation of *cis*-acting elements; Table S3: The primers used in this study.
