# Supplementary material for: Identification of the Gossypium hirsutum SDG Gene Family and Functional Study of GhSDG59 in Response to Drought Stress
Source: Plants (Basel). 2024 Apr 30;13(9):1257. doi: 10.3390/plants13091257 (PMC11085088; doi:10.3390/plants13091257)
Supplement: Supplementary file 1 [file plants-13-01257-s001.zip › Suplementary figure.pdf]

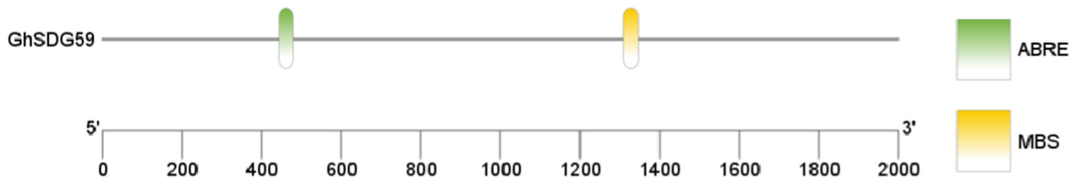

Figure S1. Analysis of *cis*-acting elements on *GhSDG59* gene promoter

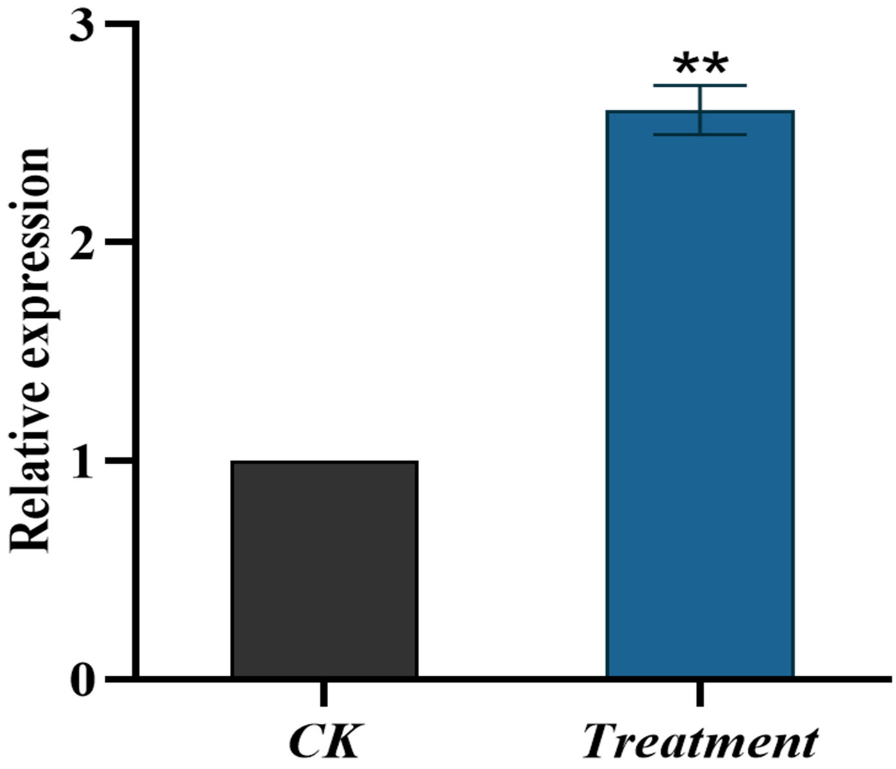

Figure S2. Expression analysis of the *GhSDG59* gene under normal watering and drought stress. The *GhHis3* as an internal reference gene for qPCR experiment. The error bar represents the standard deviation of three independent biological replicates. The asterisks indicate significant differences according to student's t-test. *, $p < 0.05$; **, $p < 0.01$

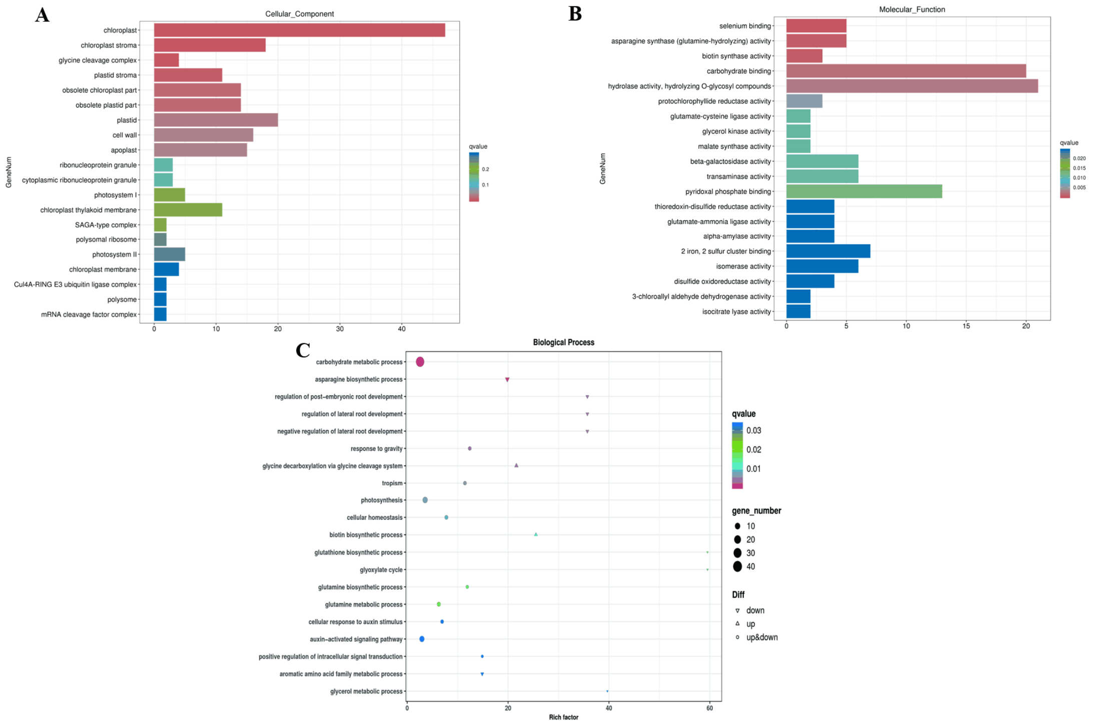

Figure S3. Gene Ontology annotation analysis of DEGs. (**A**) DEGs are annotated to cellular components. The histogram shows the number of genes for each category. (**B**) DEGs are annotated to molecular functions. The histogram shows the number of genes for each category. (**C**) DEGs are annotated to biological processes. The histogram shows the number of genes for each category.
