# Supplementary material for: Identification of the Gossypium hirsutum SDG Gene Family and Functional Study of GhSDG59 in Response to Drought Stress
Source: Plants (Basel). 2024 Apr 30;13(9):1257. doi: 10.3390/plants13091257 (PMC11085088; doi:10.3390/plants13091257)
Supplement: Supplementary file 1 [file plants-13-01257-s001.zip › Suplementary table.pdf]

Table S1. Characteristics of SDG family genes in *Gossypium hirsutum*

Gene name	Sequence ID	Number of Amino Acid	Molecular Weight	Theoretical pI	Instability Index	Hydrophilicity coefficient	Subcellular localization
GhSDG1	Ghir_A01G001580	327	37112.26	4.69	57.73	-0.225	Cytoplasm
GhSDG2	Ghir_A02G007430	2299	249781.71	5.49	43.8	-0.698	Nucleus
GhSDG3	Ghir_A02G007840	479	54508.24	8.14	43.67	-0.184	Cytoplasm
GhSDG4	Ghir_A02G009870	1245	138061.71	8.51	43.71	-0.488	Nucleus
GhSDG5	Ghir_A02G017730	1502	169863	6.17	44	-0.507	Nucleus
GhSDG6	Ghir_A02G017740	1503	169871.58	5.85	46.3	-0.517	Nucleus
GhSDG7	Ghir_A03G006100	427	49065.01	9.37	53.78	-0.441	Mitochondria
GhSDG8	Ghir_A03G016960	866	97874.12	9.21	39.71	-0.833	Nucleus
GhSDG9	Ghir_A03G018020	2427	276053.45	6.11	50.65	-0.79	Nucleus
GhSDG10	Ghir_A05G024300	1732	190774.11	6.64	49.61	-0.594	Nucleus
GhSDG11	Ghir_A05G029100	657	72891.73	8.57	44.66	-0.498	Nucleus
GhSDG12	Ghir_A05G034670	701	78218.91	9.09	40.44	-0.407	Chloroplast
GhSDG13	Ghir_A05G041020	664	73333.98	7.08	35.86	-0.43	Nucleus
GhSDG14	Ghir_A05G042680	1093	123910.38	8.59	48.42	-0.543	Nucleus
GhSDG15	Ghir_A06G011840	625	69646.04	6.16	51.36	-0.132	Nucleus
GhSDG16	Ghir_A06G012070	492	55990.92	5.63	42.29	-0.689	Nucleus
GhSDG17	Ghir_A07G003450	1560	171890.48	8.63	48.65	-0.511	Nucleus
GhSDG18	Ghir_A07G017550	357	41011.27	8.84	50.52	-0.22	Extracel
GhSDG19	Ghir_A08G005450	239	27193.25	8.15	47.17	-0.397	Nucleus
GhSDG20	Ghir_A08G009060	561	64097.16	4.95	40	-0.218	Nucleus
GhSDG21	Ghir_A08G017250	1038	115708.41	5.45	50.73	-0.725	Nucleus
GhSDG22	Ghir_A08G020070	726	80642.21	5.52	50.1	-0.539	Mitochondria

GhSDG23	Ghir_A09G013870	337	37014.59	6.65	60.84	0.026	Chloroplast
GhSDG24	Ghir_A09G024510	693	77642.06	5.41	48.6	-0.323	Nucleus
GhSDG25	Ghir_A09G024920	390	43134.72	4.47	55.38	-0.117	Chloroplast
GhSDG26	Ghir_A10G013820	1042	117726.77	7.27	39.41	-0.468	Nucleus
GhSDG27	Ghir_A11G014170	829	92676.32	5.96	57.1	-0.511	Nucleus
GhSDG28	Ghir_A11G020600	940	104825.16	8.73	50.74	-0.698	Nucleus
GhSDG29	Ghir_A11G024460	371	42436.92	9.31	57.26	-0.619	Mitochondria
GhSDG30	Ghir_A11G025060	360	39894.34	5.56	54.71	-0.217	Nucleus
GhSDG31	Ghir_A11G033330	697	76963.84	6.88	46.45	-0.459	Nucleus
GhSDG32	Ghir_A12G005330	445	49931.1	5.59	32.51	-0.25	Nucleus
GhSDG33	Ghir_A12G012070	1019	116396.3	7.83	47.08	-0.584	Chloroplast
GhSDG34	Ghir_A12G013390	748	83780.3	6.31	51.01	-0.847	Nucleus
GhSDG35	Ghir_A12G016310	475	52536.95	6.04	34.32	-0.008	Chloroplast
GhSDG36	Ghir_A12G022660	919	103086.92	8.36	51.17	-0.597	Nucleus
GhSDG37	Ghir_A12G026340	1091	124448.12	8.79	46.89	-0.59	Nucleus
GhSDG38	Ghir_A13G000150	667	74096.13	8.4	40.12	-0.485	Nucleus
GhSDG39	Ghir_A13G010790	457	51796.77	6.48	44.04	-0.59	Cytoplasm
GhSDG40	Ghir_A13G021830	398	45749.26	8.66	51.58	-0.356	Cytoplasm
GhSDG41	Ghir_D01G001580	483	54356.85	4.92	52.52	-0.284	Nucleus
GhSDG42	Ghir_D02G007880	2316	251988.18	5.31	44.54	-0.7	Nucleus
GhSDG43	Ghir_D02G008260	482	54808.69	7.4	44.46	-0.14	Cytoplasm
GhSDG44	Ghir_D02G010380	1256	139068.43	8.4	45.44	-0.525	Nucleus
GhSDG45	Ghir_D02G018230	969	109444.2	9.52	39.4	-0.841	Nucleus
GhSDG46	Ghir_D02G019330	2055	234437.15	6.66	53.65	-0.818	Nucleus

GhSDG47	Ghir_D03G001810	1538	173683.23	6.01	44.9	-0.495	Nucleus
GhSDG48	Ghir_D03G001820	1534	173641.2	6.1	44.99	-0.511	Nucleus
GhSDG49	Ghir_D03G011650	879	98941.54	5.23	46.53	-0.453	Nucleus
GhSDG50	Ghir_D03G012790	327	36920.68	9.13	56.71	-0.494	Mitochondria
GhSDG51	Ghir_D04G000200	1059	119992.82	8.6	50.77	-0.572	Nucleus
GhSDG52	Ghir_D04G001950	664	73041.78	7.07	34.87	-0.399	Nucleus
GhSDG53	Ghir_D04G006380	443	49944.88	9.31	53.52	-0.223	Chloroplast
GhSDG54	Ghir_D04G007810	701	77965.44	8.98	40.65	-0.431	Chloroplast
GhSDG55	Ghir_D05G024150	1970	217207.93	7.94	50.17	-0.634	Nucleus
GhSDG56	Ghir_D05G029170	657	72758.62	8.64	45.06	-0.5	Nucleus
GhSDG57	Ghir_D06G012440	492	56052	5.58	42.93	-0.7	Nucleus
GhSDG58	Ghir_D06G012650	420	46119.41	5.73	57.55	-0.106	Nucleus
GhSDG59	Ghir_D06G020680	429	48337.95	8.46	44.18	-0.221	Cytoplasm
GhSDG60	Ghir_D07G003460	2063	226969.43	8.48	48.29	-0.461	Nucleus
GhSDG61	Ghir_D08G005570	202	22773.99	5.33	42.43	-0.339	Nucleus
GhSDG62	Ghir_D08G018070	808	90503.49	7.23	48.95	-0.61	Nucleus
GhSDG63	Ghir_D08G020910	726	80803.42	5.53	51.01	-0.549	Nucleus
GhSDG59	Ghir_D09G013370	337	37108.6	6.18	59.59	0.018	Cytoplasm
GhSDG65	Ghir_D09G023660	634	71116.54	5.65	49.73	-0.369	Nucleus
GhSDG66	Ghir_D09G024110	390	42978.48	4.55	55.78	-0.139	Chloroplast
GhSDG67	Ghir_D10G013790	1063	120076.5	8.2	39.74	-0.483	Nucleus
GhSDG68	Ghir_D11G014210	757	84236.75	6.08	57.89	-0.531	Nucleus
GhSDG69	Ghir_D11G020640	921	102800.8	8.74	51.82	-0.733	Nucleus
GhSDG70	Ghir_D11G024200	333	38139.86	8.67	46.1	-0.545	Cytoplasm

GhSDG71	Ghir_D11G025210	362	39902.21	5.94	53.13	-0.259	Nucleus
GhSDG72	Ghir_D11G026160	150	16730.86	8.97	34.39	-0.431	Cytoplasm
GhSDG73	Ghir_D11G036710	697	76636.5	6.39	45.93	-0.434	Nucleus
GhSDG74	Ghir_D12G005300	363	41863.09	5.66	39.15	-0.058	Chloroplast
GhSDG75	Ghir_D12G012280	1019	116300.14	7.92	46.19	-0.593	Chloroplast
GhSDG76	Ghir_D12G013620	882	98522.42	7.17	46.34	-0.754	Nucleus
GhSDG77	Ghir_D12G016540	362	40389.91	6.26	35.72	-0.036	Golgi apparatus
GhSDG78	Ghir_D12G022640	919	103145.8	8.16	52.67	-0.614	Nucleus
GhSDG79	Ghir_D12G026360	1091	124625.38	8.75	45.92	-0.581	Nucleus
GhSDG80	Ghir_D13G000350	667	74160.13	8.48	42.12	-0.511	Nucleus
GhSDG81	Ghir_D13G011430	432	48882.68	7.66	42.6	-0.548	Mitochondria
GhSDG82	Ghir_D13G022570	489	55759.89	8.93	49.78	-0.38	Nucleus

Table S2. Function annotation of *cis*-acting elements

**Cis-acting element name**	**Function annotation**
ABRE	cis-acting element involved in the abscisic acid responsiveness
MBS	MYB binding site involved in drought-inducibility

Table S3. The primers used in this study

**Primer**	**Sequence (5'-3')**
GhSDG59-VIGS-F	CATCATCACCTGGTTCTTC
GhSDG59-VIGS-R	CACATAAGTAGAAATTGGT
GhSDG59-qPCR-F	GTTACGACAGTCGGGTACGGTG
GhSDG59-qPCR-R	GCAACGGTCGATACAAGCAACC
GhHIS3-qPCR-F	GCGCAAAGGTTGGTGTCTTC
GhHIS3-qPCR-R	TCAAGACTGATTTGCGTTTCCA
